# A long non-coding RNA that harbors a SNP associated with type 2 diabetes regulates the expression of *TGM2* gene in pancreatic beta cells

**DOI:** 10.3389/fendo.2023.1101934

**Published:** 2023-02-07

**Authors:** Itziar González-Moro, Henar Rojas-Márquez, Maialen Sebastian-delaCruz, Jon Mentxaka-Salgado, Ane Olazagoitia-Garmendia, Luis Manuel Mendoza, Aina Lluch, Federica Fantuzzi, Carmen Lambert, Jessica Ares Blanco, Lorella Marselli, Piero Marchetti, Miriam Cnop, Elías Delgado, José Manuel Fernández-Real, Francisco José Ortega, Ainara Castellanos-Rubio, Izortze Santin

**Affiliations:** ^1^ Department of Biochemistry and Molecular Biology, University of the Basque Country UPV/EHU, Leioa, Spain; ^2^ Biocruces Bizkaia Health Research Institute, Barakaldo, Spain; ^3^ Department of Genetics, Physical Anthropology and Animal Physiology, University of the Basque Country, Leioa, Spain; ^4^ Institut d’Investigació Biomèdica de Girona, Girona, Spain; ^5^ CIBER Fisiopatología de la Obesidad y Nutrición (CIBERobn), Instituto de Salud Carlos III, Madrid, Spain; ^6^ ULB Center for Diabetes Research, Université Libre de Bruxelles, Brussels, Belgium; ^7^ Health Research Institute of the Principality of Asturias (ISPA), Oviedo, Spain; ^8^ University of Barcelona, Barcelona, Spain; ^9^ Endocrinology and Nutrition Department, Central University Hospital of Asturias (HUCA), Oviedo, Spain; ^10^ Department of Medicine, University of Oviedo, Oviedo, Spain; ^11^ Department of Clinical and Experimental Medicine, Cisanello University Hospital, Pisa, Italy; ^12^ Division of Endocrinology, Erasmus Hospital, Université Libre de Bruxelles, Brussels, Belgium; ^13^ Spanish Biomedical Research Network in Rare Diseases (CIBERER), Madrid, Spain; ^14^ Department of Medical Sciences, School of Medicine, University of Girona, Oviedo, Spain; ^15^ Diabetes and Associated Metabolic Diseases Networking Biomedical Research Centre, Madrid, Spain; ^16^ Ikerbasque - Basque Foundation for Science, Bilbao, Spain

**Keywords:** long non-coding RNA, type 2 diabetes, single nucleotide pholymorphism (SNP), pancreatic beta cell, transglutaminase 2

## Abstract

**Introduction:**

Most of the disease-associated single nucleotide polymorphisms (SNPs) lie in non- coding regions of the human genome. Many of these variants have been predicted to impact the expression and function of long non-coding RNAs (lncRNA), but the contribution of these molecules to the development of complex diseases remains to be clarified.

**Methods:**

Here, we performed a genetic association study between a SNP located in a lncRNA known as LncTGM2 and the risk of developing type 2 diabetes (T2D), and analyzed its implication in disease pathogenesis at pancreatic beta cell level. Genetic association study was performed on human samples linking the rs2076380 polymorphism with T2D and glycemic traits. The pancreatic beta cell line EndoC-bH1 was employed for functional studies based on LncTGM2 silencing and overexpression experiments. Human pancreatic islets were used for eQTL analysis.

**Results:**

We have identified a genetic association between LncTGM2 and T2D risk. Functional characterization of the LncTGM2 revealed its implication in the transcriptional regulation of TGM2, coding for a transglutaminase. The T2Dassociated risk allele in LncTGM2 disrupts the secondary structure of this lncRNA, affecting its stability and the expression of TGM2 in pancreatic beta cells. Diminished LncTGM2 in human beta cells impairs glucose-stimulated insulin release.

**Conclusions:**

These findings provide novel information on the molecular mechanisms by which T2D-associated SNPs in lncRNAs may contribute to disease, paving the way for the development of new therapies based on the modulation of lncRNAs.

## 1 Introduction

Type 2 diabetes (T2D) is a complex metabolic disease that develops in genetically susceptible individuals (1). Indeed, the trigger of T2D development is presumed to be a combination of lifestyle and environmental factors working together with the genetic background (2). Genome-wide association studies (GWAS) have identified several genomic regions associated with the risk of T2D (3). Although these studies have provided a better understanding of T2D genetics, most of the genetic variants identified so far fall into non-coding regions of the genome. The molecular mechanism by which these variants increase risk of T2D remains to be clarified.

Transglutaminase 2 (TGM2) is a calcium-dependent multifunctional enzyme that can act as GTPase or transamidase, and that participates in several cellular processes, including apoptosis, cell adhesion or insulin release, among others (4). Disruption of *TGM2* in mice has been associated with increased glucose levels, and reduced insulin release in response to glucose (5). In addition, missense mutations in *TGM2* have been associated with early onset T2D and maturity onset diabetes of the young (MODY) (6).

A recent study identified a lncRNA (*LOC107987281* or *LncTMG2*) located within the first intron of the *TGM2* gene. The same study revealed that the expression of the lncRNA was tightly correlated with the expression of the *TGM2* coding gene in several cell lines and tumor tissues, suggesting its role as a cis acting transcriptional regulatory lncRNA (7).

LncRNAs are non-coding RNA molecules of more than 200 nucleotides in lenght that participate in several cellular and biological processes, including transcriptional regulation (8). Most of the complex disease-associated variants are located in non-coding regions of the human genome, and more specifically, in lncRNAs. The presence of disease-associated single nucleotide polymorphism (SNPs) in exonic regions of lncRNAs usually disrupt their secondary structure, affecting their capacity to interact with other macromolecules, and eventually altering their function (9). Although the function of most lncRNAs has not been annotated yet, there is already accumulating evidence of their implication in the development of several diseases, including metabolic disorders (10–12).

In the present work, we have described a genetic association between a SNP located in the coding sequence of *LncTGM2* and T2D and related traits. In addition, we have characterized the relation between *LncTGM2* and *TGM2* in pancreatic beta cells and unveiled the mechanisms by which *LncTGM2* might induce beta cell dysfunction in T2D.

## 2 Materials and methods

### 2.1 Association study

Cohort 1 consisted of 725 individuals (47 ± 11 years, 54% men) recruited in the northwest of Spain, including general population, and obesity and diabetes outpatient clinics in which the percentage of obese individuals was 72% and the percentage of type 2 diabetic individuals was 11% (13). Cohort 2 included 616 Caucasian subjects selected for a study of non-classic cardiovascular risk factors performed in the northwest of Spain (Asturias) (14). Participants (52 ± 12 years, 45% men, 26% obesity, 11% T2D) were randomly identified from a census and invited to participate.

Clinical characterization of human cohorts included a standardized questionnaire, physical examination and the performance of routine laboratory tests. Height and weight were measured by trained personnel using calibrated scales and a wall-mounted stadiometer, respectively, and with the participant in light clothing and without shoes. Body mass index (BMI) was calculated by dividing weight in kilograms by the square of the height in meters (kg/m^2^). Obesity was set at BMI≥30 kg/m^2^. The waist of the subjects was measured with a soft tape midway between the lowest rib and the iliac crest, hip circumference was measured at the widest part of the gluteal region, and waist-to-hip ratio was then calculated. Together with clinically relevant information and subsidiary data, the number of cigarettes/day (if any) and the use of hormonal contraceptives were recorded. In those participants that agree (>75%), oral glucose tolerance test (OGTT) was performed to measure glucose tolerance. Blood samples from all the participants were collected, and after 15 minutes, tubes were centrifuged at 4,000 r.p.m. at room temperature. The serum and peripheral blood leukocytes were separated and immediately frozen at –80°C. Genomic DNA was extracted from blood samples following standard purification methods (QIAamp DNA Blood Mini Kit, Qiagen, Hilden, Germany) and DNA quantity and purity was determined using a spectrophotometer (GeneQuant, GE Health Care, Piscataway, USA). The targeted single nucleotide polymorphism (SNP) rs2076380 was genotyped by means of a predesigned rhAmp™ allelic discrimination assay (Hs.GT.rs2076380.A.1; Thermo Fisher Scientific, Massachusetts, USA) and the rhAmp Genotyping Master Mix (IDT, Coralville, USA), using a LightCycler 480 RT-qPCR System sequence detector (Roche Diagnostics, Barcelona, Spain). Replicates and positive and negative controls were included in all reactions.

### 2.2 Cell cultures and human cDNA samples

The EndoC-βH1 human pancreatic cell line (Univercell Biosolutions, Paris, France) was cultured in plates coated with Matrigel-fibronectin (100 mg/ml and 2 mg/ml, respectively; Sigma-Aldrich, Burlington, USA) in Opti-β1 medium (Univercell Biosolutions). DMEM containing 5.6 mmol/l glucose, 2% vol/vol Fetal Bovine Serum, 50 μmol/l 2-mercaptoethanol (Bio-Rad, Hercules, USA), 10 mmol/l nicotinamide (Calbiochem, Darmstadt, Germany), 5.5 μg/ml transferrin and 6.7 ng/ml selenite (Sigma-Aldrich) was used for transfection.

EndoC-βH1 cell line was Mycoplasma free as determined by the MycoAlert Mycoplasma Detection kit (Lonza). For the prevention of Mycoplasma contamination, Plasmocin Prophylactic (Invivogen, Toulouse, France) was added to the culture medium on a regular basis.

cDNA samples from human pancreatic islets were obtained from Cisanello University Hospital, Pisa, Italy. All the islets were isolated and cultured using the same experimental conditions and following established isolation procedures (15). Characteristics of islet preparations are described in **Table S1**. The Ethical Committee of Cisanello University Hospital approved experiments using human islets.

### 2.3 Silencing experiments

*LncTGM2* silencing in the EndoC-βH1 cell line was performed by transfecting 30 nmol/l of a siRNA targeting *LncTGM2* (CD.Ri.214258.13.13, IDT) using Lipofectamine RNAimax reagent (Thermo Fisher Scientific) following the manufacturer’s instructions.

### 2.4 Plasmid construction and transfection

For overexpressing plasmids, *LncTGM2* was purchased as a gBlock (IDT) and cloned into a modified pCMV6 vector using KpnI and FseI restriction enzymes (New England Biolabs, Ipswich, USA). Plasmids were transfected using Lipofectamine 2000 Transfection Reagent (Invitrogen, Carlsbad, USA) following the manufacturer’s instructions.

### 2.5 Cell treatments

EndoC-βH1 cells were exposed to Actinomycin D (Sigma-Aldrich) at a final concentration of 5 μg/ml for 2, 4 or 6h. Palmitate treatment was performed by adding BSA-palmitic acid (0.5 mmol/l; 1:1) to DMEM/F-12, complemented with 0.25% vol/vol FBS, 50 μmol/l 2-mercaptoethanol (Bio-Rad), 10 mmol/l nicotinamide (Calbiochem), 5.5 μg/ml transferrin, 6.7 ng/ml selenite (Sigma-Aldrich), 100 units/ml penicillin and 100 μg/ml streptomycin (Lonza) for 4 or 8h.

### 2.6 Cellular fractionation

For *LncTGM2* RNA quantification in subcellular fractions of EndoC-βH1 cells, nuclei were isolated using C1 lysis buffer (1.28 mol/l sucrose, 40 mmol/l Tris -HCl pH 7.5, 20 mmol/l MgCl_2_, 4% vol/vol Triton X-100). *LncTGM2*, *MEG3* (nuclear control) and *RPLP0* (cytoplasmic control) expression levels were measured by RT-qPCR and compared to the total amount of those RNAs in the whole cell lysate.

### 2.7 RNA isolation and RT-qPCR

RNA extraction was performed using the NucleoSpin RNA Kit (Macherey Nagel, Düren Germany) and expression values were determined by RT-qPCR using iTaq Universal SYBR Green Supermix (Bio-Rad) using specific primers for each target RNA (**Table S2**). All RT-qPCR measurements were performed in duplicates and expression levels were analyzed using the 2^–ΔΔCt^ method. A commercially available RNA panel set (Human total RNA master panel II, Clontech, Saint-Germain-en-Laye, France) was used to assess *LncTGM2* and *TGM2* expression levels in different human tissues.

### 2.8 Western blot analysis

EndoC-βH1 cells were washed with cold PBS and lysed in Laemmli buffer (62 mmol/l Tris-HCl, 100 mmol/l dithiothreitol (DTT), 10% vol/vol glycerol, 2% wt/vol SDS, 0.2 mg/ml bromophenol blue, 5% vol/vol 2-mercaptoethanol). Proteins in the lysate were separated by SDS-PAGE. After electrophoresis, proteins were transferred to nitrocellulose membranes using a Transblot-Turbo Transfer System (Bio-Rad) and blocked in 5% wt/vol non-fatty milk diluted in TBST (20 mmol/l Tris, 150 mmol/l NaCl and 0.1% vol/vol Tween 20) at room temperature for 1h. The membranes were incubated overnight at 4°C with a primary antibody specific for TGM2 (15100-1-AP, Proteintech Group, Rosemont, USA) diluted 1:1000 in 5% wt/vol BSA or anti-α-tubulin (Cat #T9026, Sigma-Aldrich) diluted 1:5000 in 5% wt/vol BSA. Immunoreactive bands were revealed using the Clarity Max Western ECL Substrate (Bio-Rad) after incubation with a horseradish peroxidase-conjugated anti-rabbit (1:1000 dilution in 5% wt/vol non-fatty milk) or anti-mouse (1:5000 dilution in 5% wt/vol non-fatty milk) secondary antibody for 1h at room temperature. The immunoreactive bands were detected using a Bio-Rad Molecular Imager ChemiDoc XRS and quantified using ImageLab software (Bio-Rad).

### 2.9 *TGM2* promoter reporter assay

*TGM2* promoter sequence was cloned into an empty pBV-Luc plasmid (Addgene, Watertown, USA) using KpnI and EcoRI restriction enzymes. EndoC-βH1 cells were transfected with a control vector (ovCTRL) or a vector overexpressing *LncTGM2* (ov*LncTGM2*), and co-transfected with the *TGM2* promoter reporter vector plus a pRL-CMV plasmid (used as an internal control) using Lipofectamine 2000 Transfection Reagent (Invitrogen). Dual-Luciferase Reporter Assay System (Promega, Madison, USA), was used to measure bioluminescence following the manufacturer’s protocol.

### 2.10 *In silico* secondary structure prediction

Secondary structure of *LncTGM2* harboring the different alleles of rs2076380, rs7275079 and rs2067027 SNPs was predicted using the RNAsnp Web Server tool (16).

### 2.11 RNA mobility shift assay

*LncTGM2* harboring rs2076380-A or rs2076380-G alleles were *in vitro* transcribed using T7 RNA Polymerase kit (TaKaRa, Kusatsu, Japan). RNAs were run in a native TBE 2% wt/vol agarose gel and migration profile was analyzed in a ChemiDoc XRS apparatus (Bio-Rad).

### 2.12 Insulin release

For insulin release experiments, *LncTGM2*-silenced EndoC-βH1 were left in Opti-β2 (Univercell Biosolutions) starving medium for 24h. After glucose starvation, cells were incubated in KREBS medium (Univercell Biosolutions) for 1h, and consecutively exposed to 0 or 20 mmol/l glucose for 40 minutes. Supernatant and lysate were harvested and insulin release and content measured by a commercial human insulin ELISA kit (Mercodia, Uppsala, USA) according to the manufacturer’s instructions.

### 2.13 Statistics

The association between the rs2076380 single variation in the *TGM2* gene, clinical parameters and the risk of T2D was assessed using SPSS Statistics (IBM). Departures from Hardy-Weinberg equilibrium were tested in all groups using a chi-square goodness of fit test with one degree of freedom. The risk of developing T2D under exposure to rs2076380 *TGM2* genotypes was evaluated using logistic regression to estimate Odd Ratios (OR), considering a dominant model in which G-allele carriers (i.e., AG-heterozygotes plus GG-homozygotes) were the reference group. To compare groups with respect to continuous variables, one-way ANOVA for multiple comparisons was used. Other statistical tests and plots were performed using GraphPad Prism 8 software (Dotmatics). Significance-level was set at p-value <0.05. Results for *in vitro* functional studies are represented as means ± standard error of mean (S.E.M.).

## 3 Results

### 3.1 An exonic SNP in *LncTGM2* is associated with T2D risk

In order to determine the potential association of *LncTGM2* with T2D clinical parameters, we performed an association study by genotyping a SNP located in the exonic region of *LncTGM2* (rs2076380; chr20:38,165,027-38,165,227, hg38). This SNP can be considered as a tagSNP since it is in high linkage disequilibrium (LD>0.8) with other SNPs in the region (**Figure S1**). The *LncTGM2* SNP rs2076380 was tested in association with measures of T2D and other metabolic and clinical parameters in two independent cohorts (**Table S3**). In cohort 1, the frequency of AA-individuals for the *LncTGM2* SNP was 8.6%, similarly to the observed frequency in Cohort 2 (8.3%). These frequencies are in line with the observed frequency of the minor allele (A) in Caucasian populations (1000 Genomes Europe; A allele frequency = 0.32) (17) and Spanish control individuals (Medical Genome Project healthy controls from Spanish population; A allele frequency = 0.225) (18).

**Figure 1:**
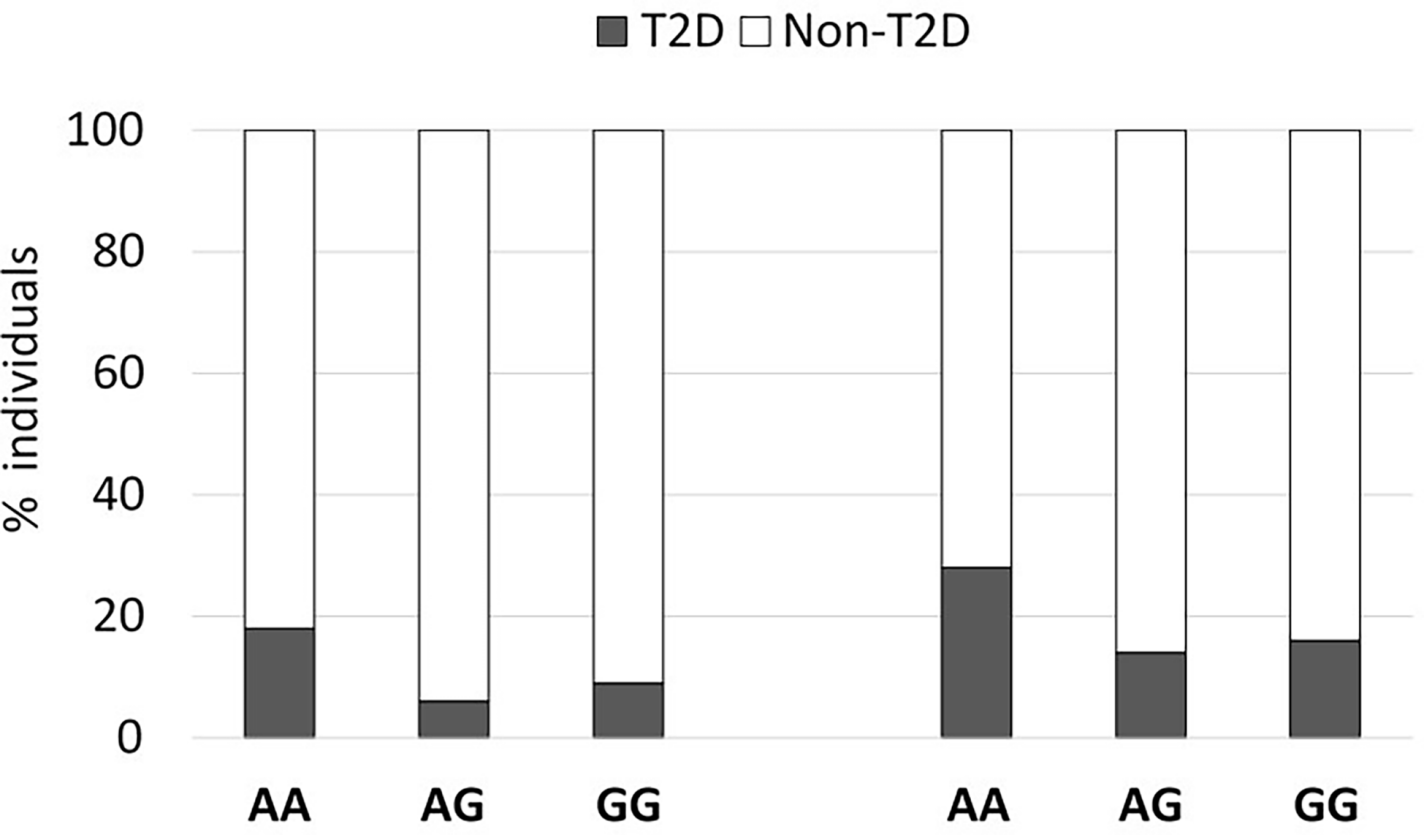
An exonic SNP in *LncTGM2* is associated with T2D risk. The graph shows the percent of T2D in two independent cohorts segregated according to their rs2076380 genotype.

As observed in **Figure 1**, the percentage of known type 2 diabetic individuals was increased in individuals harboring the rs2076380-AA genotype in both cohorts (Cohort 1: OR=1.13 [0.999-1.27], Pearson’s Chi-square p=0.006, two-sided Fisher’s exact test p=0.013); and Cohort 2: OR=1.08 [0.996-1.18], Pearson’s Chi-square p=0.018, two-sided Fisher’s exact test p=0.026). For both cohorts, regression analyses depicted the impact of the polymorphism in *LncTGM2* on T2D incidence (ANOVA p-value of 0.026 in Cohort 1, and p=0.013 in Cohort 2) after correcting for sex and age. A codominant genetic model that included age, weight and sex effects was fitted to estimate the ORs between the exposure to the AA, AG and GG genotypes, the later as the reference group. The similar ORs for AG and GG genotypes obtained for the codominant model suggested the possibility of fitting a recessive model for AA-genotype carriers. This model allowed us to determine the OR between carriers of the AA genotype in relation to the G-allele porters. In this case, the residual deviance of the genotype, once age, weight and sex were added to the model, reached a p-value <0.05, indicating that the genotype effect was significant.

In addition, we observed that in Cohort 1, fasting glucose (p=0.005) and insulin levels (p=0.006) were increased compared to G-allele carriers (**Table S3**). However, in Cohort 2, association with fasting glucose only reached statistical significance in female participants (**Table S3**).

### 3.2 *LncTGM2* expression is correlated with *TGM2* expression in several human tissues and regulated by lipotoxicity in pancreatic beta cells

**Figure 2:**
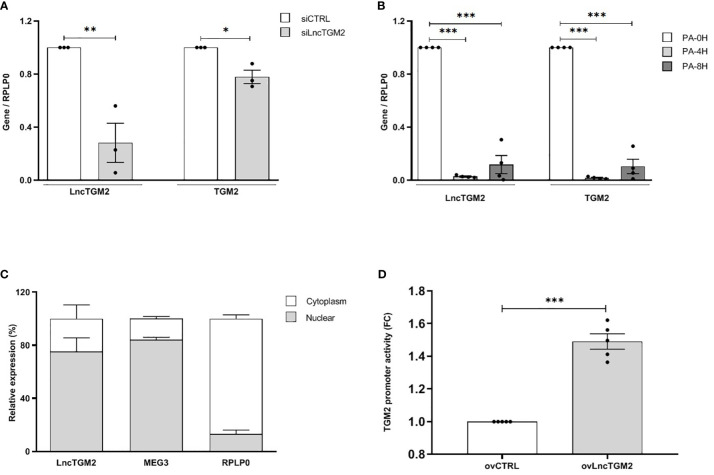
*LncTGM2* co-expresses with *TGM2* and regulates its transcriptional activity in pancreatic beta cells. **(A)** *LncTGM2* was silenced in the EndoC-βH1 cell line using a siRNA, and **(B)** EndoC-βH1 cells were exposed to palmitate (0.5 mM) for 4 or 8h. *LncTGM2* and *TGM2* expression was assessed by RT-qPCR and normalized by the reference gene *RPLP0*. The results are means ± S.E.M. of 3-4 independent experiments; *p < 0.05, **p < 0.01, and ***p < 0.001 by Student’s t-test. **(C)** RT-qPCR analysis of *LncTGM2*, *MEG3* (as nuclear marker) and *RPLP0* (as cytoplasmic marker) in nuclear and cytoplasmic fractions in EndoC-βH1 cells. **(D)** HEK-293 cells were transfected with a control vector (ovCTRL) or a vector overexpressing *LncTGM2* (ov*LncTGM2*), and co-transfected with a *TGM2* promoter luciferase reporter construct plus a pRL-CMV plasmid (used as internal control). After 48h of recovery, bioluminescence was measured.

Previous studies have correlated *LncTGM2* and *TGM2* expression in tumor tissues and some human cell lines, including lymphoblast (K562), promyeoloblast (HL60) and monocyte (THP-1) cell lines (7). In order to clarify whether *LncTGM2* and *TGM2* expression was also correlated in healthy human tissues and in pancreatic beta cells, we first evaluated the expression of both genes in EndoC-βH1 cells and a set of human tissues. The highest expression of both, *LncTGM2* and *TGM2*, was found in lung, placenta and heart, and the expression in the EndoC-βH1 cell line was similar to that of intestine and liver (**Figure S2**). Spearman’s correlation analysis showed a significant correlation between *LncTGM2* and *TGM2* expression across the tissues analyzed (R=0.87 (0.59-0.9); p<0.0001). Interestingly, a correlation was also seen in EndoC-βH1 cells using siRNA-driven inhibition of *LncTGM2*. As shown in **Figure 2A**, a 70% decrease of *LncTGM2* expression reduced *TGM2* mRNA expression by 20%, suggesting a potential implication of *LncTGM2* in the transcriptional regulation of *TGM2*.

In order to simulate the pathophysiological conditions of T2D in pancreatic beta cells, we next exposed EndoC-βH1 cells to palmitate (PA) as an *in vitro* model of lipotoxicity (19). As shown in **Figure 2B**, 4 and 8h PA exposure decreased both *LncTGM2* and *TGM2* expression in EndoC-βH1 cells, suggesting that in the presence of a lipotoxic insult the expression of both genes is reduced.

### 3.3 *LncTMG2* regulates the transcriptional activity of *TGM2*

Knowledge of the subcellular localization of lncRNAs is crucial to understand and characterize their function. In contrast to protein-coding mRNAs, lncRNA themselves should be located in their site of action, and thus, their location within the cell is crucial for their function. While nuclear lncRNAs are usually implicated in the regulation of transcriptional activity, cytoplasmic lncRNAs can participate for example, in the regulation of mRNA stability or in protein translation (20). Having this in mind, we next decided to analyze the subcellular localization of *LncTGM2* in EndoC-βH1 cells. As shown in **Figure 2C**, *LncTGM2* was detected in both nuclear and cytoplasmic fractions, but its expression level was significantly higher in the nuclear compartment, suggesting its potential implication in transcriptional regulation. Since expression of *LncTGM2* and *TGM2* was significantly correlated in pancreatic beta cells, we performed a promoter reporter assay to clarify whether *LncTGM2* was directly regulating the promoter activation of *TGM2* gene. To this aim, we constructed an expression vector coding for a luciferase under the control of the promoter of *TGM2*. The luciferase vector was then co-transfected in EndoC-βH1 cells with an empty overexpression plasmid (ovCTRL) or with the overexpression plasmid of *LncTGM2* (ov*LncTGM2*) and the activation of *TGM2* promoter was determined by measuring bioluminiscence. As shown in **Figure 2D**, the activation of the *TGM2* promoter was 1.5-fold higher in *LncTGM2*-overexpressing cells than in control cells, pointing out a role of *LncTGM2* in the activation of *TGM2* promoter, and consequently in the transcriptional activation of *TGM2*.

### 3.4 The T2D-associated risk allele in *LncTGM2* disrupts its secondary structure impacting on its stability, and correlates with decreased expression of *TGM2* in beta cells

**Figure 3:**
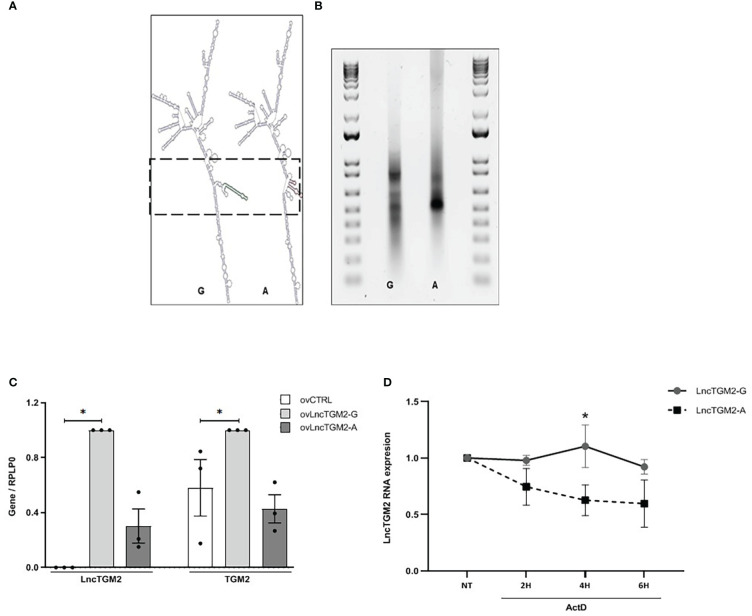
The T2D-associated risk allele in *LncTGM2* disrupts its secondary structure impacting on its stability, and correlates with decreased expression of *TGM2* in beta cells. (A) *In silico* prediction of the secondary structure of *LncTGM2* harboring each allele for rs2076380; T2D protective allele (G) or T2D risk allele **(A)**. **(B)** Electrophoretic mobility profiles of *in vitro*-transcribed *LncTGM2* molecule harboring the T2D protective allele (rs2076380-G) or the risk allele (rs2076380-A). **(C)** EndoC-βH1 cells were transfected with overexpression plasmids of *LncTGM2* harboring the protective (ov*LncTGM2*-G) or risk allele (ov*LncTGM2*-A) for T2D, and mRNA levels of *LncTGM2* and *TGM2* were determined by RT-qPCR and normalized to *RPLP0.* The results are means ± S.E.M. of 3 independent experiments; *p < 0.05 by Student’s t-test. **(D)** EndoC-βH1 cells were transfected with *LncTGM2* overexpression plasmids harboring the protective (ov*LncTGM2*-G) or risk allele (ov*LncTGM2*-A) for T2D. EndoC-βH1 cells were exposed to Actinomycin D (ActD) (5 μg/ml) for 2, 4 or 6h and *LncTGM2* mRNA level was determined by RT-qPCR. The results are means ± S.E.M. of 3 independent experiments. *p < 0.05 ov*LncTGM2*-G vs. ov*LncTGM2*-A at the same time-point.

Disease-associated SNPs located within lncRNAs can affect their function through the disruption of their secondary structure (21, 22). As previously shown (**Figure S1**), the T2D-associated rs2076380 SNP is in high LD with other two SNPs located in the exonic region of *LncTGM2* (rs7275079 and rs2067027). To assess whether these SNPs alter the secondary structure of *LncTGM2*, we performed an *in silico* prediction analysis using the RNAsnp webserver from the Center for non-coding RNA in Technology and Health (23). Interestingly, rs2076380 was predicted to significantly alter the secondary structure of *LncTGM2* (p=0.0803), while the software did not predict any significant change in the structure of the lncRNA when the different alleles of rs7275079 or rs2067027 SNPs were present (p>0.2) (data not shown). As shown in **Figure 3A**, the predicted secondary structures of *LncTGM2* carrying the T2D protective (rs2076380-G) or risk allele (rs2076380-A) were significantly different. Consistent with the prediction, *in vitro*–transcribed forms of T2D protective and risk allele–harboring *LncTGM2* revealed different motilities on a native agarose gel (**Figure 3B**), suggesting a different conformation of the lncRNA in the presence of one or other allele in rs2076380.

Taking into account that the secondary structure of a lncRNA is crucial for its interaction with other macromolecules, and thus, for its function (9), we next decided to determine whether the genotype of the T2D-associated SNP in *LncTGM2* affected *TGM2* expression in human pancreatic islets. To this aim we genotyped rs2076380 SNP and measured *TGM2* expression in 16 cDNA samples from human islets, and performed an eQTL analysis. As shown in **Figure S3**, there was a trend for higher expression of *TGM2* in islets harboring the protective rs2076380-GG genotype compared to islets harboring the risk allele in heterozygosis (rs2076380-AG) or homozygosis (rs2076380-AA), although the differences did not reach statistical significance, probably due to the limited number of islets.

Next, to characterize the potential effect of each allele in rs2076380 SNP on the expression of both, *LncTGM2* and *TGM2*, we constructed two *LncTGM2* overexpression plasmids, one harboring the T2D risk allele (ov*LncTGM2*-A), and the other harboring the T2D protective allele (ov*LncTGM2*-G). Interestingly, allele-specific upregulation of *LncTGM2* in beta cells revealed that the expression level reached by transfecting ov*LncTGM2*-G plasmid was higher than the expression level obtained with ov*LncTGM2*-A plasmid (**Figure 3C**), suggesting that the T2D risk allele might be affecting the stability of *LncTGM2* RNA molecule.

In order to directly test whether the T2D-associated polymorphism affected *LncTGM2* stability, we next performed an allele-specific overexpression of *LncTGM2* and exposed the EndoCβ-H1 cells to Actinomycin D, a drug that inhibits transcription. As shown in **Figure 3D**, *LncTGM2* harboring the protective allele (rs2076380-G) was more stable than the lncRNA harboring the risk allele (rs2076380-A) at all time-points, although the differences only reached statistical significance at 4h of Actinomycin D treatment (p<0.05). These results confirmed that the *LncTGM2* risk allele in the T2D-associated rs2076380 SNP reduced the stability of the lncRNA.

To clarify whether the decreased stability of *LncTGM2*-A affected its capacity to regulate *TGM2* expression, we next analyzed the expression of *TGM2* in EndoC-βH1 cells overexpressing *LncTGM2*-A or *LncTGM2*-G. As observed in **Figure 3C**, only the upregulation of the lncRNA harboring the protective allele (ov*LncTGM2*-G) increased the expression of *TGM2* mRNA. These results were also confirmed at the protein level (**Figure S4**).

In summary, these results suggested that the *LncTGM2* harboring the T2D risk allele induced less *TGM2* expression due to its reduced stability.

### 3.5 *LncTGM2* downregulation affects glucose-stimulated insulin secretion

Previous studies have shown that TGM2 might be implicated in insulin release through different mechanisms, including cytoplasmic actin remodeling and regulation of the action of other proteins during granule movement (24). Taking into account that our present results suggest that the T2D risk allele in *LncTGM2* might induce a decrease in *TGM2* expression in pancreatic beta cells, we next decided to determine the potential contribution of *LncTGM2* in insulin release.

**Figure 4:**
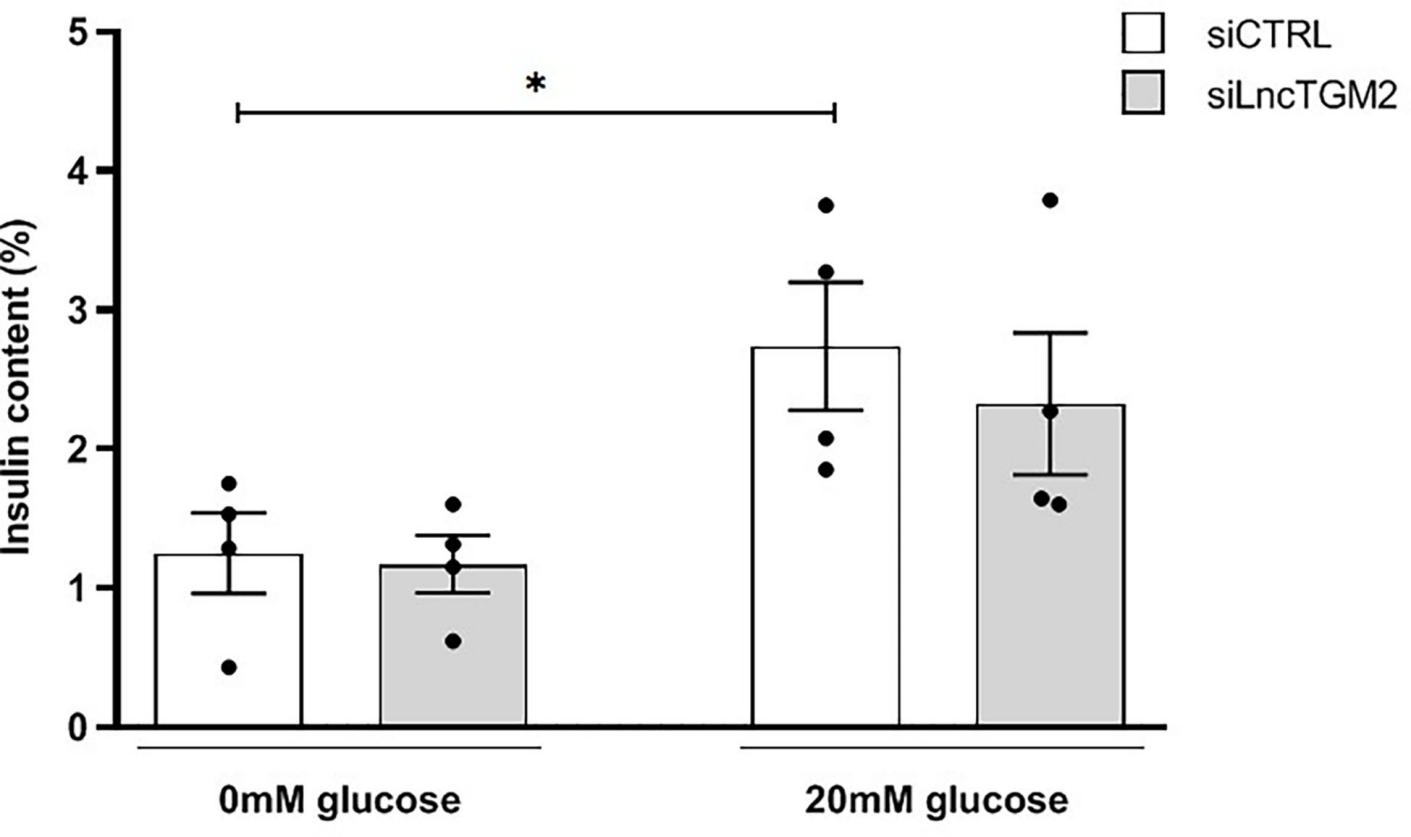
*LncTGM2* downregulation affects glucose-stimulated insulin secretion. *LncTGM2* was silenced using a siRNA and EndoC-βH1 cells were exposed to 0 or 20 mmol/l glucose. Insulin release was determined by ELISA. The results are means ± S.E.M. of 4 independent experiments; *p<0.05 by Student’s t-test.

To this aim, we silenced *LncTGM2* with a specific siRNA in EndoC-βH1 cells and determined glucose-stimulated insulin release. As shown in **Figure 4**, high glucose stimulation in siCTRL-transfected EndoC-βH1 cells increased insulin secretion. In si*LncTGM2*-transfected beta cells, however, high glucose-induced insulin secretion (GSIS) was no longer statistically significant, suggesting that disruption of *LncTGM2* in pancreatic beta cell might affect GSIS through diminished expression of TGM2.

## 4 Discussion

In the current study, we identified a genetic association between *LncTGM2* and T2D and glycemic traits in two independent cohorts. Previous GWAS in larger Caucasian populations have not detected a genetic association between rs2076380 and T2D, however based on phenotype-wide association data (T2D knowledge portal), this polymorphism has been associated with T2D-related complications (e.g. microalbuminuria). Moreover, based on the T2D knowledge portal, the genomic region in which *LncTGM2* is located (also containing *TGM2*, *RPRD1B* and *KIAA1755* genes) has been associated with several metabolic and glycemic traits, including cardiovascular disease related parameters, cholesterol and type 2 diabetes. The main reason for the discordance between our findings and GWAS data may lie on the fact that our two cohorts are enriched for obese individuals (especially Cohort 1), and in our both cohorts, T2D incidence seem to be associated with obesity (data not shown). In this sense, several studies have described a link between TGM2 and obesity and associated glycemic traits. For example, a study found that loss of TGM2 sensitizes for diet-induced obesity-related inflammation and insulin resistance (25). Moreover, a network-based approach to assess the cellular processes associated with protein–protein interaction subnetworks of glycemic traits showed that TGM2 was associated with both, HOMA-β and HOMA-IR, suggesting a potential role of this protein in pancreatic beta cell function and insulin resistance (26). The same study concluded that HOMA-β-associated GWAS genes (which include *TGM2*) enriched pathways of fat metabolism, especially in adipose tissues, supporting the “lipotoxicity theory” of beta cell failure in T2D.

In line with this hypothesis, in the present study, we have observed a co-expression between *LncTGM2* and the coding gene *TGM2* in pancreatic beta cells under basal and lipotoxic conditions. Our data suggest that lipotoxicity, a typical feature of obesity-associated T2D, reduces *LncTGM2*, which in turn provokes a reduction of *TGM2* in pancreatic beta cells. Indeed, lipotoxicity (e.g. high fat diet) has been previously associated with *TGM2* expression reduction in other tissues, including liver (27).

Moreover, we propose a mechanism by which *LncTGM2* may affect glucose-stimulated insulin release through *TGM2* expression reduction in an allele-specific manner. The lncRNA *LncTGM2* lies within the first intron of the *TGM2* gene (9), which encodes a multifunctional enzyme that has been implicated in the pathogenesis of early onset T2D and MODY (6). Interestingly, early onset T2D and MODY-associated *TGM2* mutants have altered enzymatic activities, such as reduced transamidation and kinase activity that impact in glucose-stimulated insulin release (28). Transcriptional regulation of *TGM2* is controlled by several transcription factors, including nuclear factor-kappa B, RA receptor/retinoid X receptor, liver X receptor and Sp1 (4). Here, we show for the first time that *LncTGM2* participates in the transcriptional regulation of *TGM2* in pancreatic beta cells. We observed that the T2D-associated risk allele in *LncTGM2* correlates with a reduction of *TGM2* expression in pancreatic beta cells. Moreover, our results suggest that a reduction in *TGM2* expression in human beta cells impair glucose-stimulated insulin release. These observations are in line with studies in rodents, in which reduced TGM2 activity has been linked to impaired glucose-stimulated insulin secretion (GSIS) (28), and also with data showing that naturally occurring mutations altering TGM2 enzymatic activities correlate with reduced insulin secretion (29). Interestingly, TGM2 has also been shown to interact with nuclear proteins (e.g. BAF and H3) immediately upon a glucose stimulus, suggesting that it may be involved not only in insulin secretion, but also in the regulation of glucose-induced gene transcription (30).

Although the molecular mechanisms by which *LncTGM2* participates in the regulation of *TGM2* transcription remain to be fully clarified, our results demonstrate that a T2D-associated polymorphism affects the secondary structure of the lncRNA, and, eventually, disrupts its function. Several other disease-associated SNPs that alter the secondary structure of lncRNAs affect the regulation of genes that participate in important pathways for disease pathogenesis, including type 1 diabetes and cardiovascular disease (31, 32). Here we demonstrate that the T2D risk allele in *LncTGM2* reduces its stability, affecting *TGM2* expression in pancreatic beta cells. Some studies have suggested that disease-associated SNPs in lncRNAs may affect RNA-turnover through disruption of the binding of proteins that regulate stability, and thus, affecting their biological function (33–35).

In conclusion, our results show that *LncTGM2* is associated with T2D and suggest that it might be implicated in disease pathogenesis through an allele-specific downregulation of *TGM2* in pancreatic beta cells. Our findings provide new information on the molecular mechanisms by which T2D-associated SNPs in lncRNAs cause disease and open the door to the development of novel diagnostic tools and therapeutic approaches based on lncRNA modulation.

## Data availability statement

The original contributions presented in the study are included in the article/**Supplementary Materials**. Further inquiries can be directed to the corresponding authors.

## Author contributions

IS and AC-R conceived and designed the study. FO and AL performed the genetic association studies. ED, JF-R, CL and JA-B coordinated human samples, clinical information, written consents and intellectual content collection. LM and PM provided the human pancreatic islet material. HR-M, IG-M, JM-S, MS-C, AO-G, FF, LM-M and MC designed and performed the experimental procedures. HR-M and IG-M wrote the paper. IS and AC-R reviewed the manuscript. All authors contributed to the article and approved the submitted version.
