# Supplementary material for: A long non-coding RNA that harbors a SNP associated with type 2 diabetes regulates the expression of TGM2 gene in pancreatic beta cells
Source: Front Endocrinol (Lausanne). 2023 Feb 7;14:1101934. doi: 10.3389/fendo.2023.1101934 (PMC9941620; doi:10.3389/fendo.2023.1101934)
Supplement: Supplementary file 1 [file DataSheet_1.pdf]

Supplementary Material

1. Supplementary Figures

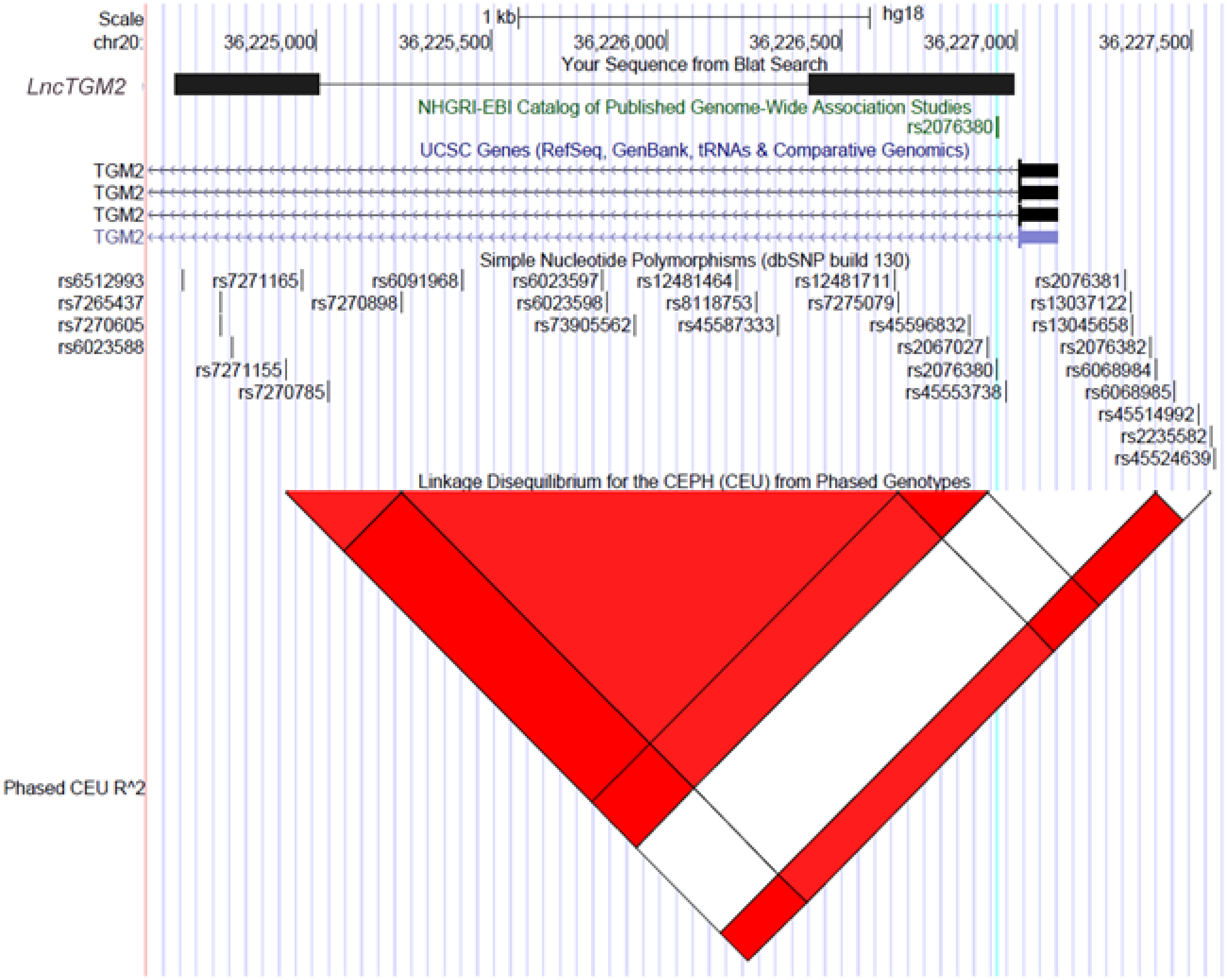

Figure S1: Genomic context of *LncTGM2* gene. The figure shows *LncTGM2* localization within *TGM2* gene. Haplotype block shows SNPs in high linkage disequilibrium (r2>0.8) with the T2D-associated SNP rs2076380 (highlighted with the blue line).

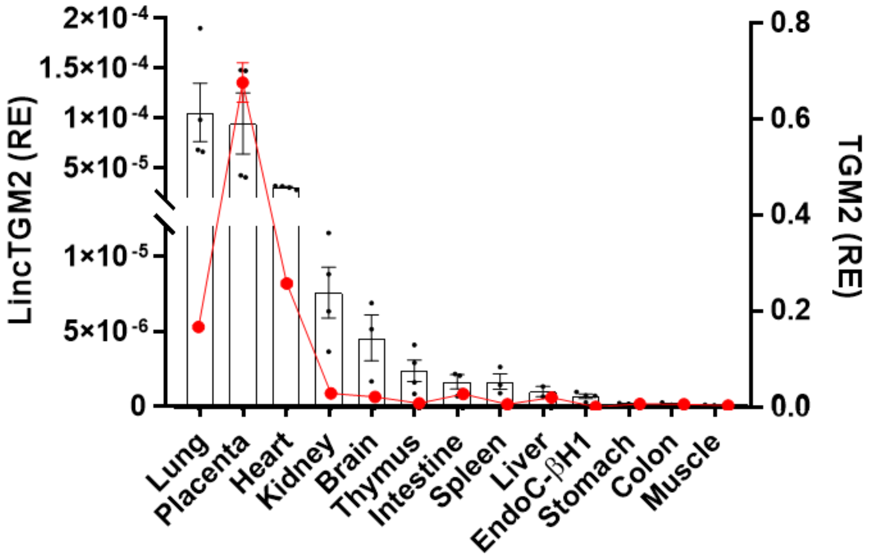

Figure S2: Expression of *LncTGM2* and *TGM2* correlate in different human tissues. *LncTGM2* (white bars) and *TGM2* (red dots) gene expression was assessed by RT-qPCR in the EndoC-βH1 cell line and in a set of human tissues (lung, placenta, heart, kidney, brain, thymus, intestine, spleen, liver, stomach, colon and muscle). The correlation analysis between the expression of both genes was performed using a Spearman correlation test (R= 0.87(0.59-0.9); p<0.0001).

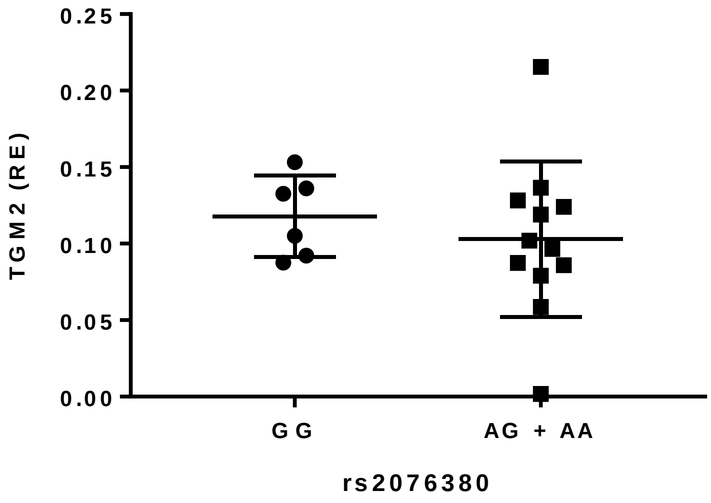

Figure S3: *TGM2* expression tends to be higher in human pancreatic islets from individuals harboring the protective T2D genotype in *LncTGM2*. eQTL analysis correlating rs2076380 genotype and *TGM2* gene expression in human pancreatic islets. Results are means ± SEM of 6 samples with the homozygous protective genotype (GG) and 12 samples harboring the risk allele (AG + AA).

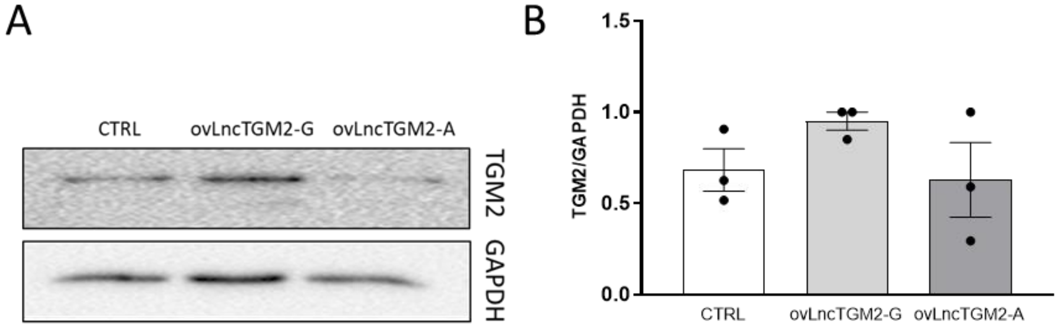

Figure S4: Upregulation of *LncTGM2* harboring the protective allele correlates with higher amounts of TGM2 protein in beta cells. (A) *LncTGM2* was overexpressed in the EndoC-βH1 cell line using specific plasmids for *LncTGM2* harboring the T2D protective (G) or risk allele (A). TGM2 protein expression was determined by Western blot. GAPDH was used as a loading control. The results are representative of 3 independent experiments. (B) Densitometry results for TGM2 protein quantification represented as means ± SEM of 3 independent experiments.

2. Supplementary Tables

Table S1. Characteristics of human pancreatic islets preparations. All the islets were collected and isolated in Cisanello University Hospital, Pisa.

**Islet preparation**	**1**	**2**	**3**	**4**	**5**	**6**
Unique identifier	23/29	23/54	23/87	23/32	23/34	21/31
Donor age (years)	77	83	59	71	72	73
Donor sex (M/F)	F	M	M	F	F	M
Donor BMI (kg/m^2)	31.25	24.97	28.73	31.22	22.89	24.69
Donor HbA or other measure of blood glucose control (mg/dl)	182	121	211	158	156	114
Donor history of diabetes?	No	No	No	No	No	No
Donor cause of death	Cardiovascular disease	Cardiovascular disease	Cardiovascular disease	Cardiovascular disease	Cardiovascular disease	Cardiovascular disease
Cold ischaemia time (h)	16	13	16	18	18	17
Glucose-stimulated insulin secretion or other functional measurement	Glucose-stimulated insulin secretion	Glucose-stimulated insulin secretion	Glucose-stimulated insulin secretion	Glucose-stimulated insulin secretion	Glucose-stimulated insulin secretion	Glucose-stimulated insulin secretion
**Islet preparation**	**7**	**8**	**9**	**10**	**11**	**12**
Unique identifier	21/80	21/94	22/37	22/40	22/57	22/95
Donor age (years)	76	66	74	78	77	59
Donor sex (M/F)	F	F	F	F	F	M
Donor BMI (kg/m^2)	17.1	24.4	29.3	24.98	19.53	25.88

Donor HbA or other measure of blood glucose control (mg/dl)	157	158	159	NA	194	118
Donor history of diabetes?	No	No	No	No	No	No
Donor cause of death	Trauma	Cardiovascular disease	Cardiovascular disease	Cardiovascular disease	Cardiovascular disease	Cardiovascular disease
Cold ischaemia time (h)	17	NA	17	17	19	31
Glucose-stimulated insulin secretion or other functional measurement	Glucose-stimulated insulin secretion	Glucose-stimulated insulin secretion	Glucose-stimulated insulin secretion	Glucose-stimulated insulin secretion	No	No
**Islet preparation**	**13**	**14**	**15**	**16**	**17**	**18**
Unique identifier	22/99	22/111	22/115	23/13	23/17	23/27
Donor age (years)	77	79	79	51	87	73
Donor sex (M/F)	F	F	F	M	M	F
Donor BMI (kg/m^2)	31.25	18.37	23.12	23.15	21.6	27.06
Donor HbA or other measure of blood glucose control (mean plasma glucose during Intensive Care Unit stay – mg/dl)	143	132	NA	93	100	153
Donor history of diabetes?	No	No	No	No	No	No
Donor cause of death	Trauma	Cardiovascular disease	Cardiovascular disease	Cardiovascular disease	Cardiovascular disease	Cardiovascular disease
Cold ischaemia time (h)	12	18	15	15	12	18

Glucose-stimulated insulin secretion or other functional measurement	Glucose-stimulated insulin secretion	Glucose-stimulated insulin secretion	Glucose-stimulated insulin secretion	No	Glucose-stimulated insulin secretion	Glucose-stimulated insulin secretion

Table S2. List and sequences of the primers used for RT-qPCR.

Gene	Primer	Sequence (5’-3’)
LncTGM2	Forward	GACCTTGGCCAATTGTTGAG
LncTGM2	Reverse	AGTTTCAGATGGAGCAGAGGAC
TGM2	Forward	CAAGGCCCGTTTTCCACTAAG
TGM2	Reverse	GAGGCGATACAGGCCGATG
MEG3	Forward	TGAAGAACTGCGGATGGAAG
MEG3	Reverse	CACGTAGGCATCCAGGTGAT
RPLP0	Forward	GCAGCATCTACACCCTGAAG
RPLP0	Reverse	CACTGGCAACATTGCGGAC

Table S3. Characteristics and clinical parameters of subjects included in the genetic association study stratified by rs2076380 genotype. Values show mean ± S.D. BMI: body mass index, S/DBP: systolic/diastolic blood pressure, OGTT: oral glucose tolerance test, L/HDL: low/high density proteins, w: women. Obesity was set at BMI≥30 kg/m^2. "Chi-square" refers to Pearson Chi-Square, and "Fisher" for two-tailed Fisher's exact test when comparing the AA-group to the G-allele carriers (AG plus GG genotypes) in non-parametric tests. Normal distribution and homogeneity of the variances were evaluated using Levene's test, and One-way ANOVA was applied to continuous variables when comparing the three groups. Italics depict significant results (95% confidence interval).

**Cohort 1**	**a. AA (n=62)**	**b. AG (n=321)**	**c. GG (n=342)**	**Chi-Square**	**Fisher**
Sex (% men)	53	56	53	ns	ns
Known diabetes (%)	18	6	9	*0.006*	*0.013*
Obesity (%)	71	71	74	ns	ns
**Clinical parameters**				**ANOVA**	**ANOVA (w)**
Age (years)	50±11	46±11	46±11	*0.020*	0.091
BMI (kg/m^2)	37.9±9.2	38.6±10.1	38.9±10	0.754	0.843
Waist-to-hip ratio	0.89±0.26	0.83±0.37	0.83±0.42	0.436	0.397
SBP (mmHg)	140.2±23.2	135.2±20.1	134.3±19.2	0.106	*0.017*
DBP (mmHg)	78.1±10.4	77.5±12.2	77.4±13.3	0.925	0.42
OGTT; glucose 0 min	102.3±24.5	95.9±15.6	96±19.3	*0.032*	*0.005*
OGTT; glucose 120 min	135.7±48.1	128.3±41.7	128.9±40.1	0.501	0.171
Insulin: 0 min	16.9±21.3	13.4±10.2	13.7±15.4	0.243	*0.006*
Insulin: 120 min	74.5±60.1	77.3±63.6	71.6±57.7	0.543	0.081
HOMA-B	138.4±128	154.9±130.2	159.8±164.5	0.624	0.985
HOMA-IR	4.71±9.75	3.38±3.41	3.47±5.27	0.199	*0.04*
Glycated haemoglobin (%)	5.62±0.68	5.50±0.56	5.52±0.74	0.431	0.078

Cholesterol (mg/dl)	205.5±36.3	195.7±35.4	195.2±36.9	0.109	*0.036*
HDL Cholesterol (mg/dl)	51.7±10.6	51.4±14.4	49.3±12.9	0.104	0.149
LDL Cholesterol (mg/dl)	131.4±32	120.6±30.9	122.7±32	0.05	*0.002*
Triglycerides (mg/dl)	113.1±48.8	118.6±73.3	114.2±59.3	0.637	0.124
**Cohort 2**	**a. AA (n=51)**	**b. AG (n=278)**	**c. GG (n=287)**	**Chi-Square**	**Fisher**
Sex (% men)	44	48	42.2	ns	ns
Known diabetes (%)	28	14	16	*0.018*	*0.026*
Obesity (%)	28	22	27	ns	ns
**Clinical parameters**				**ANOVA**	**ANOVA (w)**
Age (years)	52±13	51±13	53±12	0.246	0.574
BMI (kg/m^2)	27.9±5.4	27.3±4.5	27.4±4	0.618	0.327
Waist-to-hip ratio	0.85±0.08	0.88±0.09	0.88±0.08	0.111	0.388
SBP (mmHg)	138±25	135.2±22.1	136.2±21.9	0.678	0.917
DBP (mmHg)	78.6±9.9	79.3±10.7	79.6±9.8	0.824	0.696
OGTT; glucose 0 min	101.9±29	98.9±24.3	98.1±25.9	0.076	*0.023*
OGTT; glucose 120 min	121.8±53.6	110.1±48.9	118.4±50.7	0.34	0.978
Insulin: 0 min	8.73±6.58	9.34±8.34	8.84±5.29	0.655	0.999
HOMA-IR	2.36±2.14	2.45±2.68	2.2±1.58	0.404	0.784
Glycated haemoglobin (%)	4.92±0.86	4.79±0.74	4.83±0.93	0.619	0.499
Cholesterol (mg/dl)	219.6±38.3	208.9±37.0	211.1±35.8	0.158	0.343
HDL Cholesterol (mg/dl)	61±18	58.8±14.6	58.5±15.5	0.554	0.55

LDL Cholesterol (mg/dl)	137.5±33.1	128.2±33.7	130.3±32.0	0.183	0.259
Triglycerides (mg/dl)	105.8±52.5	114±85	111.1±60.7	0.73	0.317
